# Microbial Degradation of Cellulosic Material and Gas Generation: Implications for the Management of Low- and Intermediate-Level Radioactive Waste

**DOI:** 10.3389/fmicb.2019.00204

**Published:** 2019-02-13

**Authors:** Danielle Beaton, Phillip Pelletier, Richard R. Goulet

**Affiliations:** ^1^Chalk River Laboratories, Canadian Nuclear Laboratories, Chalk River, ON, Canada; ^2^Orillia Soldiers’ Memorial Hospital, Ottawa, ON, Canada; ^3^Canadian Nuclear Safety Commission, Ottawa, ON, Canada

**Keywords:** microbial cellulose degradation, low- and intermediate-level radioactive waste, gas generation, pressure, methanogenesis, acetogenesis

## Abstract

Deep geologic repositories (DGR) in Canada are designed to contain and isolate low- and intermediate-level radioactive waste. Microbial degradation of the waste potentially produces methane, carbon dioxide and hydrogen gas. The generation of these gases increase rock cavity pressure and limit water ingress which delays the mobility of water soluble radionuclides. The objective of this study was to measure gas pressure and composition over 7 years in experiments containing cellulosic material with various starting conditions relevant to a DGR and to identify micro-organisms generating gas. For this purpose, we conducted experiments in glass bottles containing (1) wet cellulosic material, (2) wet cellulosic material with compost Maker, and (3) wet cellulosic material with compost Accelerator. Results demonstrated that compost accelerated the pressure build-up in the containers and that methane gas was produced in one experiment with compost and one experiment without compost because the pH remained neutral for the duration of the 464 days experiment. Methane was not formed in the other experiment because the pH became acidic. Once the pressure became similar in all containers after 464 days, we then monitored gas pressure and composition in glass bottle containing wet cellulosic material in (1) acidic conditions, (2) neutral conditions, and (3) with an enzyme that accelerated degradation of cellulose over 1965 days. In these experiments, acetogenic bacteria degraded cellulose and produced acetic acid, which acidity suppressed methane production. Microbial community analyses suggested a diverse community of archaea, bacteria and fungi actively degrading cellulose. DNA analyses also confirmed the presence of methanogens and acetogens in our experiments. This study suggests that methane gas will be generated in DGRs if pH remains neutral. However, our results showed that microbial degradation of cellulose not only generated gas, but also generated acidity. This finding is important as acids can limit bentonite swelling and potentially degrade cement and rock barriers, thus this requires consideration in the safety case as appropriate.

## Introduction

Nuclear power plant operations produce low- and intermediate-level radioactive waste (LILW). Low-level waste is radioactive waste in which the concentration of radionuclides is above 1 Becquerel per gram and contains primarily radionuclides with half-lives shorter than or equal to 30 years. Intermediate-level waste is radioactive non-fuel waste, containing significant quantities of radionuclides with half-lives greater than 30 years (Canadian Standard Association, 2013). Nuclear power plant operators should dispose of low-level waste in shallow facilities (50 m or less below ground surface), while intermediate-level waste merits more stringent isolation and should be disposed of in deep (often 400–600 m) geological repositories (DGRs) (International Atomic Energy Agency, 2009). Six countries are currently containing and isolating mixed LILW in DGRs to protect the public and the environment until the radioactivity of the waste decays to background levels (Aikas and Anttila, 2008; Brewitz et al., 2008; Olsson et al., 2008; Powers and Holt, 2008; Woller, 2008; Choung et al., 2014). In several instances, cement backfill is added to provide additional isolation of the waste (Aikas and Anttila, 2008; Olsson et al., 2008), which creates hyper alkaline conditions in the repository. For these facilities, the safety cases for disposal of LILW relied on safety assessment models predicting that the depth of the repositories would isolate the radionuclides in the waste, and the rock formation and engineered barriers (i.e., cement backfill surrounding the waste and shaft seal) would contain the radionuclides in the waste for several hundred thousand years. These long periods of isolation and containment are necessary for radionuclides to decay to background levels and ensure the protection of humans and their environment. Considering the time scale of these model predictions, uncertainties needed to be adequately quantified so that modeling provided a sufficient margin of safety to account for these uncertainties.

Low- and intermediate-level radioactive waste typically consist of ion-exchange resins, filters, carbon steel and ample cellulosic material (Husain, 2005; Small et al., 2008, 2017). As such, biodegradation of the cellulosic material of the waste is an important consideration for proposed DGR safety cases (Arter et al., 1991; Humphreys et al., 1997; Wang and Francis, 2005). Microbial communities that utilize cellulose as the primary carbon source include nitrate, iron, manganese and sulfate reducing, methanogenic and acetogenic microbes (Pereyra et al., 2010; Lever, 2012). The complete biodegradation of the cellulosic material requires a wide phylogenetic and functional diversity in a microbial community (Berlemont and Martiny, 2015). The fungal and bacterial compositions comprise the primary (Reaction 1; Table 1) (Berlemont and Martiny, 2015) and secondary fermenters that produce the carbon dioxide, hydrogen and volatile organic acids from the solid waste (Reaction 2; Table 1) (Berlemont and Martiny, 2015). The bacterial and archaeal compositions utilize the resulting fermentation products to produce hydrogen sulfide in the case of sulfate reducers (Pereyra et al., 2010), methane (Reaction 3; Table 1) in the case of methanogens (Berlemont and Martiny, 2015), and acetic acid (Reaction 4; Table 1) in the case of acetogens (Miller and Wolin, 1995; Ravinder et al., 2001; Rabemanolontsoa et al., 2017).

**Table 1:** Reaction involved in the biodegradation of cellulose and resulting gas formation.

Microbial process	Reaction equation	Reaction #
Hydrolysis of solid cellulose into β-D-cellobiose	Cellulose → β-D-cellobiose (CH_2_O)	Reaction 1
Hydrolysis of β-D-cellobiose and fermentation	CH_2_O → organic acids + CO_2_ + H_2_	Reaction 2
Hydrogenotrophic methanogenesis	4 H_2_ + CO_2_ → CH_4_ + 2 H_2_O	Reaction 3
Acetogenesis	4 H_2_ + 2CO_2_ CO_2_ → CH_3_COOH + 2 H_2_O	Reaction 4
Aceticlastic methanogenesis	CH_3_COOH → CH_4_ + CO_2_	Reaction 5


Degradation of cellulose-based waste by fungal, bacterial and archaeal communities involves several carbohydrate active enzymes, such as the glycoside hydrolases (Berlemont and Martiny, 2015) that comprise several families (Henrissat and Bairoch, 1996). Within a microbial community, most of the members are secondary fermenters (Reaction 2, Table 1) that carry only the genes for degradation of the more easily hydrolyzed carbohydrates (Berlemont and Martiny, 2015). These include reactions catalyzed by the glycoside hydrolase families 1 and 3 (Davies and Henrissat, 1995) that degrade the carbohydrate β-D-cellobiose. Only a small fraction of the community members would be primary fermenters (Reaction 1; Table 1) that carry the genes for both cellulose and β-D-cellobiose degradation (Berlemont and Martiny, 2015).

It is challenging to estimate the length of time that cellulosic material will be available to microbial degradation because of the limited number of microbial organisms able to degrade solid cellulose. As a result, microbial degradation of solid cellulose can often be the rate limiting step. Some studies suggest cellulose and associated breakdown product β-D-cellobiose are completely degraded within 100–500 years (Pavasars et al., 2003) while other authors estimated complete degradation of cellulose at 100,000 to 10,000,000 years (VanLoon and Glaus, 1997). Glaus and Van Loon (2008) narrowed the degradation time to a best estimate of 1000–5000 years in alkaline conditions.

The rate of cellulose degradation is important to predict how a DGR will isolate and contain radionuclides in the LILW because it will influence the timing and onset of gas generation and subsequent pressure build-up over time. The sooner the gas pressure rises to equilibrium with preconstruction water pressure, the sooner water stops entering a DGR, which will dry up and remain as such as long as its gas pressure is maintained above the water pressure and as long as water is formed through methanogenesis and acetogenesis. However, if water vapor becomes limited, dry conditions could limit microbial activity, the gas pressure would slowly decrease and water will eventually resume migrating into the DGR. Then, microbial gas generation would be stimulated as long as a cellulosic substrate is available. This wet and dry cycle is likely to happen as long as cellulosic material remains available for microbial activity. As a result, during dry periods, water soluble radionuclides such as Cl-36 and I-129 would be effectively immobilized. In contrast, gaseous radionuclides such as tritium and C-14 could migrate if a pathway exists within a fault or within the excavated damage zone between the host rock and the shaft seal material. The extent of C-14 and tritium gas migration will depend on the pressure build-up in the DGR cavity. As carbon dioxide and hydrogen gas are formed, the pressure could increase and favor gaseous migration. In such situation, it should be noted that the potential migration of C-14 and tritium gases with relatively short half-lives is less of a concern because DGRs are designed to contain and isolate radionuclides with much longer half-lives. In addition, methanogenesis is assumed to decrease gas pressure by consuming both carbon dioxide and hydrogen, thereby decreasing the likely migration of C-14 and tritium gas. Hence, ideal conditions to contain water soluble and gaseous radionuclides are at a pressure that limits water ingress but also limits gaseous radionuclide migration.

Therefore, it is important to determine how microbial degradation of cellulosic material will affect the gas composition and pressure in a DGR. As there is currently no underground laboratory or DGR in Canada, we initiated laboratory experiments over a 7 years timeframe. The objective of these laboratory studies was to measure gas pressure and composition, as well as to identify microbial groups and functions associated with the production of gas.

## Materials and Methods

### Experimental Approach

To test whether methane can be the terminal gas produced within a repository isolating and containing LILW, we conducted 6 un-replicated experiments using surrogate cellulosic material. Initial starting conditions were either without or with commercial compost additives to accelerate the evolution of gases. We first monitored gas pressure and composition in two experiments with commercial compost additives and in an additional experiment without commercial compost additives (control) to investigate the effect of additives on gas pressure and composition. Once pressure build-up stabilized after 464 days, we initiated new experiments in which we monitored gas pressure and composition in one experiment with added cellulase enzymes from a fungal source, in another experiment in acidic conditions (pH 5) and a third experiment at neutral pH for an additional 1965 days.

### Sample Preparation

We used mop heads as surrogate cellulosic material because these are used for routine cleaning of nuclear facilities located within radiological zones. Approximately 100 g of mop material was used for each experiment. For the first three experiments, mop samples were wetted with sterile reverse osmosis deionized water. Mop samples were wetted to achieve at least 60% of the moisture holding capacity. The wetted samples were then inserted into separate sterile 1 L glass bottles. Before sealing the bottles, we added 0.031g of Green Cross Original Fertosan compost maker which contained 11% total nitrogen, 5% phosphoric acid, 5% soluble potash, 2% magnesium, 1% calcium, and 55% organic matter into one bottle (Compost Maker treatment). In the second bottle, we added 0.36 g of Green Earth 100% natural compost accelerator containing bone meal and char, feather meal, sulfate, potash, granular calp, and soil enzymes (Compost accelerator treatment). These two mop samples wetted with sterile water were amended to test the effects of additives on the rate of gas generation.

Following the 464 days experiments, three new experiments were initiated with new cellulosic material to test the effect of an additive and pH on gas pressure and composition. One mop sample was wetted with sterile neutral phosphate buffer (BioUltra, >0.044 M Na_2_HPO_4_ from Sigma-Aldrich) and another mop sample was wetted with citrate buffer (pH 5). The other container received 1 unit of purified cellulase enzyme from *Trichoderma viride* (lyophilized powder, 0.3–1.0 unit/mg solid, Sigma) at a starting pH of 7 using a neutral phosphate buffer. Details of the starting conditions for each experiment are listed in Table 2. These three new experiments lasted for 1965 days.

**Table 2:** Sample names, starting condition and the elapsed time in days when the headspace gases were sampled.

Experiment name	Starting condition	Headspace sampling: elapsed time in days
Control (un-amended)	No commercial compost additive added	464
Compost accelerator	Commercial blend: nutrients and enzymes	464
Compost maker	Commercial blend: nutrients and microorganisms	464
Neutral	pH 7	150, 730, and 1965
Acidic	pH 5	150, 730, and 1965
Cellulase	pH 7 with Cellulase from *Trichoderma viride*	150, 730, and 1965


### Head Space Gas Monitoring

All six containers were sealed using media bottle lids that had been modified to allow temperature and pressure gauges to be situated inside of the bottle. These penetrations were sealed with stainless steel tubing. These penetrations also provided the means for periodic sampling of the container headspace for gases. The temperature and pressure inside the containers were monitored continuously using thermocouples and differential pressure cells (Cole Parmer, 14.7–15 psig). The pressure measurements were corrected for temperature. Data from the thermocouples and pressure cells were logged automatically using a Keithley data logger. To prevent over pressurizing the containers as the mop heads degraded, each container was fitted with a 68.95 kPa (10 psi) pressure relief valve.

Table 2 lists the sample names, their starting conditions and the duration between headspace gas sampling. Three containers identified “Un-amended,” “Compost Accelerator,” and “Compost Maker” were sampled for headspace gas once, after 464 days, and then removed from continuous monitoring to start the monitoring of the other three samples. The headspace gas compositions for the neutral, acidic and cellulase experiments were determined at three time points: after 150, 730, and 1965 days of continuous monitoring. After 1965 days, all six samples were processed for gene abundances by digital drop PCR (ddPCR).

### Headspace Gas Analysis

Headspace gas analysis was performed using a Agilent 6890N gas chromatograph (GC) by connecting the pressurized sample containers (vessels) to a vacuum line, and then evacuating the line to a pressure <1 torr absolute, as measured by an MKS Baratron Capacitance Manometer. The vacuum pump was then isolated, and a sample of the headspace gas within the container was bled into the vacuum system by a low flow metering valve to a pressure of roughly 760 torr. The sample inlet was then isolated, and time was given for the pressure to stabilize in the vacuum system. This process was repeated three times on each sample to ensure sample consistency. A 100 μL sample of the analyte gas was then directed to the GC using a 6-port 2-position sampling valve. The GC was configured with an Agilent HP-PLOT Molsieve 15 m column for separation of permanent gases (Ar, O_2_, N_2_, H_2_, and CH_4_), and an Agilent GASPRO 30 m column for separation of corrosive gases (CO_2_ and H_2_S). A valve was installed to allow for column selection midway through the GC run. The analysis was performed independently using both He and Ar carrier gases for each sample to ensure detection of hydrogen gas. Detection of the gases was performed using a Thermal Conductivity Detector (TCD) and a reference gas matching the carrier (both He and Ar, respectively). Calibration of the GC was performed using certified standard mixes of the analyte gases using the same sample introduction scheme at sample pressures ranging from 10 to 760 torr. Examples of the analysis output are provided in Supplementary Figure S1.

### Leachate pH

The volume of the leachate obtained from each experiment, although sufficient for DNA analysis, was not sufficient to allow for pH measurement with conventional pH meter electrodes. Therefore, the pH was measured using pH-indicator paper. The indicator paper provided reasonable precision to compare the pH in our leachate from one experiment to the other.

### Sample Preparation for ddPCR

Following the final headspace gas sampling, the sample bottles were opened and two pieces of material approximately 1 cm in size were cut out and processed for DNA extraction using the protocol from the PowerSoil^®^ DNA Isolation kit (MoBio, 12888). DNA extracts were also obtained from liquid leachate collected from the samples using the Rapid Water DNA extraction kit. Leachate (5 ml) was first filtered through a 0.2 μm Supor polyether sulfone membrane. The extracted DNA was quantified by fluorescence using the intercalating dye, PicoGreen, from the Quant-iT PicoGreen dsDNA assay kit (Life Sciences, P7589). The detected concentration ranged from 0.08 to 0.39 ng/μL, with two samples below our detection limit (Supplementary Table S1). These extracts were stored at -20°C until they were analyzed by ddPCR.

### Primers

Microbial communities are challenging to characterize due to the phylogenetic and functional diversity required to degrade solid wastes. To gauge the cellulolytic, fermentative, sulfate-reducing, acetogenic and methanogenic microbes, a literature search was performed to find primers that targeted the genes of interest. The cellulase enzymes are involved in the early steps of cellulose degradation by catalyzing the hydrolysis of glycosidic bonds. There are several different types of cellulases based on the reaction to be catalyzed. The glycoside hydrolase family of cellulose enzymes consist of 131 protein families based on their sequence and structure information (Henrissat and Bairoch, 1996). To evaluate the potential for cellulose degradation, the set of primers used spanned genes for the exo- and endo-glucosidases that are found within bacteria and fungi (Supplementary Table S1). The primers also covered four of the families within the glycoside hydrolase enzyme class: families 4, 5, 6 within bacteria (Canizares et al., 2011), and family 61 within fungi (Busk and Lange, 2013). The primers, mlas and mcrA, are directed to the alpha-subunit of the methyl co-enzyme M reductase gene (*mcr*A) and detect methanogenic Archaea (Steinberg and Regan, 2008). This enzyme catalyzes the last step of methanogenesis, is conserved among all methanogens (Steinberg and Regan, 2008), and is absent in non-methanogens except for the anaerobic methane oxidizing Archaea (Hallam et al., 2003). We also tested the primers, dsr1-F and dsr-r (LeLoup et al., 2007), that are directed to the dissimilatory bisulfite reductase gene (*dsr*AB) as a functional marker to evaluate the sulfate reducing community (Baker et al., 2003). The dissimilar sulfate reduction trait is patchily distributed in the Tree of Life, with five bacterial and two Archaeal phyla containing recognized members of this guild; members within this guild also represent a fermentative trait. Primers for coenzyme A synthase (Aydin et al., 2015) were applied to gauge for genes involved in acetate formation from hydrogen and carbon dioxide and for genes involved in acetate consumption to form methane. Quantification of the three Kingdoms were determined using primer pairs for the 16S rRNA gene for Archaea (Baker et al., 2003), glutamine synthetase for bacteria (Hurt et al., 2001), and the 18s rRNA gene for fungi (Zhu et al., 2005).

Details of the primer sequences, targets, expected amplicon size and thermal cycler conditions are provided in Supplementary Table S1. Primers were ordered from Integrated DNA Technologies (IDT). Upon receipt, the primers were solubilized to a final concentration of 100 μM in sterile water and stored at -20°C. Multiple working stocks were diluted to 10 μM and stored at -20°C. All reagents were made up and stored in autoclaved DNase, RNase, Pyrogens, DNA and PCR inhibitor and Endotoxin free tubes. For all reactions, Sarstedt Biosphere DNA, RNase and Pyrogen free filter tips were used.

### Digital Drop PCR (ddPCR)

All ddPCR reactions were set up inside of a laminar flow hood wiped with 70% ethanol and exposed to ultraviolet light for 3 min prior to entering the hood. Individual reactions for ddPCR contained a final primer concentration of 150 nM with 2x QX200 ddPCR EvaGreen Supermix (Bio-Rad #186–4033) following manufacturer’s instructions. Two microliters of DNA were loaded into a total reaction volume of 21 μL. All ddPCR runs included a no template negative control and a positive control on some occasion. Supplementary Table S1 provides description of the positive controls and Supplementary Figure S1 provide information on positive and negative ddPCR. Twenty microliters of the ddPCR reaction were transferred to a DG8 Cartridge (Bio-Rad #186–4008) with 70 μL of Droplet Generation Oil for EvaGreen (Bio-Rad #186–4006) covered with a DG8 Gasket (Bio-Rad #186–3009) and converted to droplets with the QX200 Droplet Generator (Bio-Rad #186–4002). Droplets were then transferred to a 96-well plate (Eppendorf #0030128.575) and heat sealed at 180°C for 6.5 s with Pierceable Foil Heat Seal (Bio-Rad #1814040) using the Bio-Rad PX1 PCR Plate Sealer (#181–4000). The samples were then cycled in a Bio-Rad C1000 Touch Thermal Cycler (#185–1196) using a 2-step or a 3-step cycling program, see Supplementary Table S1. The cycled plate was then transferred and read on the QX200 reader (Bio-Rad #186-4003) and data were analyzed with the QuantaSoft Software (Bio-Rad #186-4011).

### Optimization of ddPCR

To determine the optimal concentration of DNA for ddPCR, a dilution series was constructed using two samples: one sample that displayed high expression of the target and one that displayed low expression of the target from the PCR.

To determine the optimal annealing temperature (*T*_A_) for all primers, a temperature gradient ddPCR was performed using *Glomus irregularis* gDNA at 0.1 ng/μL was used across a temperature range of 52 to 62°C in a 3-step cycle. The optimal *T*_A_ was chosen based on the highest fluorescent amplitude of the amplicon.

### Analysis of ddPCR Results

The gene abundance (concentration) was reported as copy number per μL. The positive droplets and the total accepted droplets were also reported. To fulfill Poisson’s distribution rules, samples must have more than 13,000 droplets to be analyzed. If the samples did not reach this threshold, individual samples were removed. The threshold to determine quantification of the target was set manually using samples with a well-discriminated fluorescence difference between positive and negative droplets.

### Cellulase Activity Assay

Cellulase activity within the leachate was determined after opening the sample bottles. The mop materials were removed from the bottles, and the liquid was extracted from the top, middle, and bottom portions of each sample by inserting each portion into a sterile 30 mL syringe and squeezing liquid into sterile plastic tubes. The leachates were stored at -20°C until analyzed for cellulase activity. Cellulase activity was measured in the surrogate wastes using the fluorometric-based Cellulase Activity Assay Kit (Abcam, ab189817) that detects the release of a fluorescent compound, resorufin, from a cellulase substrate, resorufin-β-D-cellobioside (Coleman et al., 2007). Detection of resorufin was by measuring the emission at 595 nm after exciting the sample at 530 nm using a Varioskan Flash Spectral Scanning Multimode plate reader (Thermo Fisher). The detected cellulase activity was compared to the cellulase activity from *T. reesei* of 6.7 × 10^-4^ μmole/mL/min (Coleman et al., 2007) as a reference. Samples having fluorescence values that exceeded the range of the standard curve were diluted and re-analyzed.

## Results

### Evolution of Gas Pressures and Composition

**FIGURE 1:**
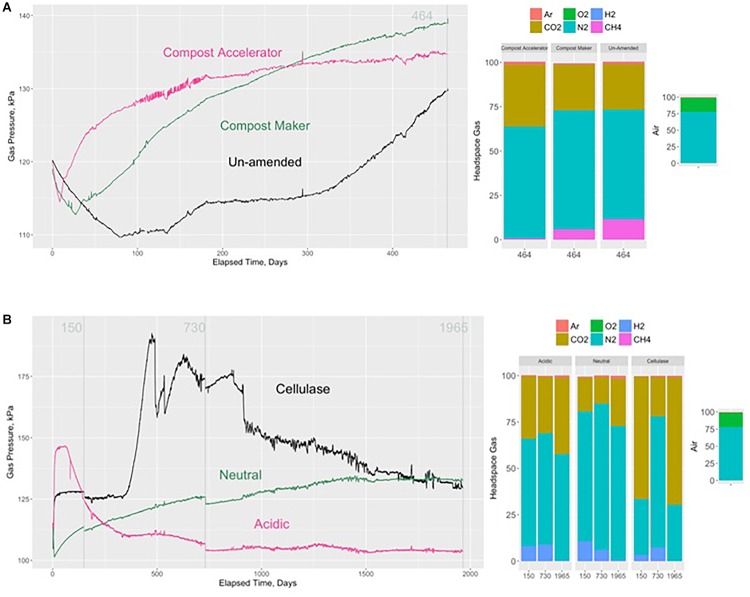
Evolution of gas pressure and headspace gas compositions without adding compost (control) and after adding Compost Accelerator and Compost Maker over 464 days **(A)**. Numbers in gray correspond to the vertical gray lines marking when the headspace gases were sampled for analysis at 464 days elapsed time. Evolution of gas pressure and headspace gas compositions after adding a citrate buffer (acidic), a phosphate buffer (neutral), and cellulase over 1965 days **(B)**. Numbers in gray correspond to the vertical gray lines marking when the headspace gases were sampled for analysis at 150, 464, 730, and 1965 days elapsed time. Also shown are the composition of the nitrogen, oxygen, argon, carbon dioxide, hydrogen, and methane in the headspaces and in dry air.

**FIGURE 2:**
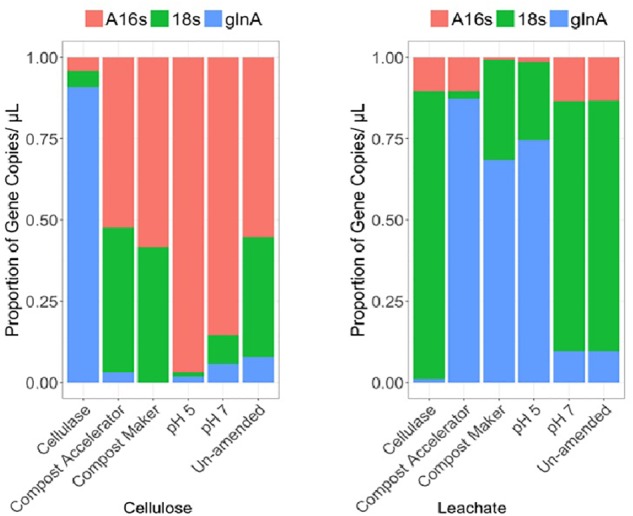
Community differences for cellulose and leachate by differential relative abundances of gene copies detected within the surrogate waste for: Archaeal rRNA (A16S), fungal rRNA (18s), and bacterial glutamine synthetase (glnA), genes.

The development of gas pressure inside each of the test containers varied in terms of the timing of gas pressure onset, the rate of gas pressure onset and the rate of gas pressure change over the monitoring period. The rate of change of pressure within the un-amended test container displayed an initial decline in gas pressure that lasted 80 days, followed by a slight increase starting at about day 130, which then leveled off at 115 kPa for the next 120 days before displaying another period of increasing pressure to 130 kPa at day 464 (Figure 1). At that time, the gas composition was 26% carbon dioxide, 62% nitrogen, 12% methane and 0.008% hydrogen (Figure 2). The pH in the leachate was pH 7.

Addition of the compost additives, Compost Maker (with microorganisms within its composition) and Compost Accelerator (with enzymes within its composition), increased the rate of gas pressure onset (Figure 1). Compared to the cellulosic material without compost added, the cellulosic material amended with the additive that included enzymes within its composition (Compost Accelerator, Figure 1) displayed the quickest onset of gas pressure and the fastest rate of gas pressure rise. These periods were preceded by an initial decline in the headspace gas pressure. By about day 50, the rate of gas pressure rise started to slow and had reached a constant level at 135 kPa by about day 250 onward to the end of the test period at 464 days. At that time, the gas composition was 35% carbon dioxide, 63% nitrogen, 0.6% methane, and 0.2% hydrogen. The pH in the leachate was at pH 4. It should be noted that as a result of low methane content, the hydrogen content in the headspace was higher.

The cellulosic material amended with the additive that included microorganisms within its composition (Compost Maker, Figure 1) displayed an initial longer decline in headspace gas pressure, a slower onset of gas pressure rise and a slower initial rate of gas pressure rise compared to the Compost Accelerator. By the end of the test period, the rate of gas pressure rise was slowing, but had not yet leveled off at 138 kPa. At that time, the gas composition was 26% carbon dioxide, 67% nitrogen, 6% methane, and 0.03% hydrogen. The pH in the leachate was between pH 6 and pH 7.

The cellulosic material with added cellulase displayed an initial rapid rate of gas pressure rise, followed by a rapid leveling off within the 1st week. Gas pressure then remained constant at 126 kPa for over 300 days. The gas pressure continued to evolve with a second rapid rise to 185 kPa followed by a period of variable pressure changes before displaying a continuous decline to similar pressure measured in the neutral experiment at day 1965. The gas composition was 66, 21, and 69% carbon dioxide, 30, 71, and 30% nitrogen, 0, 0.01, and 0% methane, and 3, 7, and 0.1% hydrogen at days 150, 730, and 1965, respectively. The pH in the leachate at the end of the experiment was at pH 3. It should be noted that hydrogen gas had depleted considerably by day 1965.

The test containers for the acidic starting condition displayed an initially rapid rate of gas pressure rise, followed by a rapid leveling off within the 1st week of starting the gas pressure monitoring, the duration of which lasted about 50 days. Afterwards, gas pressure rapidly declined to 110 kPa and remained at this pressure until the end of monitoring at day 1965 (Figure 1). At that time, the gas composition was 34, 30, and 41% carbon dioxide, 58, 60, and 57% nitrogen, 0, 0, and 0% methane, and 8, 9, and 0.5% hydrogen at days 150, 730, and 1965, respectively. The pH in the leachate at the end of the experiment was pH 6. Similar to the cellulase experiment, hydrogen gas had depleted by day 1965.

The test container with cellulosic material exposed to neutral pH conditions at the start of gas pressure monitoring displayed a gas pressure evolution that first declined then rose, followed by a rate of change that slowly leveled off over approximately 1500 days and remained unchanged at 132 kPa for the remainder of the monitoring period. The gas composition was 18, 14, and 25% carbon dioxide, 70, 79, and 72% nitrogen, 0, 0.01, and 0% methane, and 11, 6, and 0.8% hydrogen at days 150, 730, and 1965, respectively. The pH in the leachate was at pH 6. Similar to the cellulase and pH 7 experiments, hydrogen gas had depleted by day 1965.

### Glycoside Hydrolase Activity in Cellulosic Material Leachate

The results of this assay are shown in Table 3 as the cellulase activity (μmole/mL/min) and as cellulase activity relative to the maximum enzyme activity determined for a characterized cellulase from *T. reesei* of 6.7 × 10^-4^ μmole/mL/min (Coleman et al., 2007). The extracted activity leached from the cellulosic material was highest in the sample with the added cellulase (Table 3), which measured an activity three times higher than the maximum activity determined for *T. reesei*. Dilution of this leachate by 20-fold relieved some inhibition of the cellulase activity that, after dilution, displayed six to seven times higher activity than the maximum activity for *T. reesei*. The enzyme activity was still four times greater than that of *T. reesei* even after dilution by 100-fold (Table 3). The sample with the neutral starting condition and the sample amended with the Compost Maker – that included enzymes within its composition (Table 2) – each displayed between 3 and 29% of the maximum cellulase activity of *T. reesei*.

**Table 3:** Cellulase activity based on the glycoside hydrolase family 1 and 3 activities detected in the sample leachates.

Sample	Portion of the sampled extracted	Emission 595 nm	Concentration of resorufin	Cellulase enzyme activity	Fraction of enzyme activity^∗^

		RLU	nM	μmole/mL/min	%
Cellulase, 100× dilution	Top	58.6	1834 (40)	3.00 × 10^-3^	446
	Middle	60.4	1878 (23)	3.13 × 10^-3^	465
	Bottom	59.2	1824 (162)	3.04 × 10^-3^	452
Compost accelerator	Top	20.7	Not detected	–	–
	Middle	20.7	Not detected	–	–
	Bottom	20.5	Not detected	–	–
Compost maker	Top	35.0	15.2 (0.7)	2.54 × 10^-6^	0.4
	Middle	50.4	28.8	4.79 × 10^-5^	7.1
	Bottom	39.1	14.0	2.34 × 10^-5^	3.5
Acidic un-amended, pH5	Top	21.4	Not detected	–	–
	Middle	21.7	Not detected	–	–
	Bottom	21.7	Not detected	–	–
Neutral un-amended, pH7	Top	60.9	33.1 (0.2)	5.52 × 10^-5^	8
	Middle	105.4	77.1 (0.2)	1.29 × 10^-4^	19
	Bottom	152.0	118.1 (0.8)	1.97 × 10^-4^	29
Un-amended	Top	25.0	1.7 (0.3)	2.87 × 10^-6^	0.4
	Middle	24.6	1.4 (0.4)	2.28 × 10^-6^	0.3
	Bottom	23.8	0.7 (0.2)	1.10 × 10^-6^	0.2


*^∗^Values for the fraction of activity are relative to the maximum rate (*V*_*max*_) for cellulase activity from T. reesei of 6.7 × 10^-*4*^ μmole/m*L*/min (Coleman et al., 2007).Cellulase activity is based on the hydrolysis of resorufin-β-D-Cellobiose (Coleman et al., 2007).*

The two test containers whose pH was acidic [the sample with an acidic starting condition and the sample with the added Compost Accelerator (Figure 1)] each had no measured cellulase activity (Table 3).

### Quantification of Marker Genes

**FIGURE 3:**
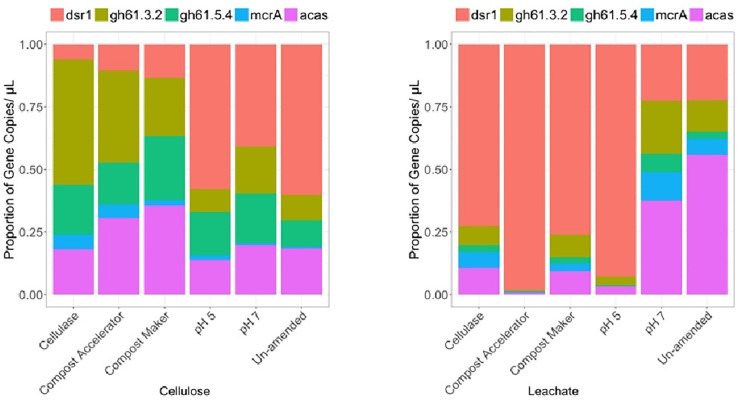
Community differences for cellulose and leachate by differential relative abundances of gene copies detected within the surrogate waste for dissimilatory sulfate reductase (dsr1), glycoside hydrolase family 61 (gh61.3.2 and gh61.5.4), methyl co-enzyme M (mcrA), and acyl co-enzyme A synthase (acas) genes.

The results for diversity of bacteria, Archaea and fungi are shown in Figure 2 and the diversity-associated functional genes are shown in Figure 3.

The Archaea were numerically dominant on the solid material across the samples except the sample amended with added cellulase (A16s, Figure 2). The gene copies representing the fungal diversity, the 18s gene (Figure 2), were higher in number than the gene copies representing the bacterial diversity, the glnA gene (Figure 2), except for the sample amended with added cellulase. In the cellulase experiment, gene copies representing the bacterial diversity, the glnA gene, made for the majority of the microbial community in that experiment, followed by the fungal and Archaea (Figure 2). Along with a more abundant fungal community on the solid cellulosic material relative to the bacteria, only the fungal gene for cellulose degradation was detected in the samples. These were represented by the primer pairs gh61.2/gh61.3 and gh61.5/gh61.4 (Figure 3) and are from the glycoside hydrolase family 61. The number of copies of these genes was approximately equal across each of the samples. Genes for the bacterial cellulolytic glycoside hydrolases were not detected, but the copies of the functional gene associated with sulfate reduction made up a large proportion of the bacterial abundance. The next most abundant genes were those genes from the acetyl co-enzyme synthetase, for aceticlastic methanogens and from the dsrAB gene for sulfate reducing bacteria and Archaea (Figure 3, acas and dsr1). The dsr1 gene copies were higher in the no compost, no compost (neutral), and no compost (acidic) samples as compared to the samples with compost (Figure 3). The containers with these additives differ in microbial composition (Figure 2, A16s, 18s,and glnA). The compost without added microbes (the cellulase treated sample) had fewer Archaea (Figure 2, A16s, red) and fungi (Figure 3, 18s, green), and had more bacteria (Figure 2, glnA, blue). Compost Accelerator and Compost Maker were most abundant in Archaea (red) and fungi (green) with relatively few bacteria (blue). The Archaea associated with methanogenesis, by the *mcr*A gene, were a minor component of all the solid samples (*mcr*A, Figure 3).

The community compositions within the leachate were different from the community compositions from the solid cellulosic material (Figure 2, 3). Overall, the marker gene for sulfate reduction, the dsrAB gene (Figure 3, dsr1) made up a large proportion of the cellulosic material without compost; in the leachate from the cellulosic material with compost; and the cellulosic material without compost that had an acidic starting condition. The phyla represented by this trait also ferment carbon and may account for some of the gas production (Figure 1). The Archaeal 16S rRNA gene (Figure 2, A16s) was present in all samples and was the main phylogenetic marker gene detected, but the marker gene for methanogens, *mcr*A (Figure 3, *mcr*A) was the lowest within all the containers. Leachate from cellulosic material amended with cellulase was dominated by fungi (Figure 2, 18s, green) rather than bacteria in the solid. In contrast; bacteria were dominant over fungi in the leachate from the compost accelerator and compost maker amendments (Figure 2, glnA, blue). All leachates had relatively fewer Archaea. The genes for fungal cellulases were detected within the leachate but the dsrAB gene was most prevalent as were the genes for coenzyme A synthetase (acas, Figure 2, pink). Overall, these results suggest that microorganisms were able to effectively degrade cellulose and β-D-cellobiose effectively except within the acidic treatment.

## Discussion

In this study, we monitored gas pressure onset, gas pressure evolution and headspace gas compositions as a result of microbial degradation of cellulose. We also tested the microbiology of the cellulosic material indirectly by measuring cellulase activity and directly by quantifying specific marker gene targets. Here, we will discuss our results, and their implications for the design of DGRs containing and isolating LILW.

### Microbial Degradation of Cellulose

The microbial degradation of cellulose is a complex process (Beguin and Aubert, 1994) that involves a number of microbial communities using a variety of enzymes to generate gases such as carbon dioxide, hydrogen, and methane. This complexity will affect the timeframe of microbial degradation. The length of time that cellulosic material will be available to microbial degradation is important to predict how a DGR will isolate and contain radionuclides in the low- and intermediate-level waste because it will influence the timing and onset of gas generation and subsequent pressure build-up over time.

In our tests, the only cellulolytic gene associated with the solid cellulosic material was from the fungal glycoside hydrolase family 61 (now called Auxiliary Activity Family 9). Copies of these genes were associated with both the solid cellulose (Figure 3, cellulose, gh61.3.2 and gh61.4.5) and the leachate (Figure 3, leachate, gh61.3.2 and gh61.4.5). These genes were present within all the containers. Their relative abundances were higher within the amended solid samples: cellulase from *T. viride*, and the commercial blends of microbes and enzymes (Compost Accelerator) and of microbes and nutrients (Compost Maker). The proportion of glycoside hydrolase family 61 marker genes (Figure 3, gh61.3.2 and gh61.4.5) was also more abundant than the marker gene for fungi (Figure 3, 18s). The microbiology of the solid waste suggests that the biodegradation is mediated by fungal cellulolytic cellulases; the bacterial cellulases, such as the cellulases from glycoside hydrolase families 1 and 3 – which were not targeted by ddPCR but were detected by enzymatic assay, would contribute to fermentation reactions that result in the hydrogen and carbon dioxide gas production.

Separately, we also detected glycoside hydrolase activity, from families 1 and 3, at various levels in the leachate (Table 3). Activity of these enzymes is responsible for the further breakdown of the small carbohydrate compounds (Batstone et al., 2002) released from the waste. The enzyme activity across the samples ranged from undetected to levels over four times the maximum velocity of a known fungal enzyme (Coleman et al., 2007). The cellulosic material amended with cellulase had the highest glycoside hydrolase activity (Table 3); inhibition of activity was relieved by dilution. The amendment of microbes and enzymes (compost accelerator, Table 3) and the un-amended cellulosic material with a starting pH of 5 had no detectable glycoside hydrolase activity and the containers between these two extremes displayed glycoside hydrolase activities from <1 to 29% of the maximum rate determined for a characterized enzyme (Table 3). The microbiology of the leachate suggests that the biodegradation of cellulosic material is aided by the fermentation by sulfate reducing prokaryotes and by the glycoside hydrolase families 1 and 3 (Table 3).

### Gas Pressure and Headspace Gases

The microbial degradation of cellulosic material generates carbon dioxide, methane, and hydrogen gas, which build up pressure in waste containers, and eventually in DGR caverns. In our tests, cellulosic material was subjected to various treatments establishing initial conditions for added microbes, enzymes and initial pH. The resulting gas evolution of pressure and composition was monitored throughout. In one set of tests, lasting 464 days, hydrogen gas was a minor component of the gas composition making up to 0.2% of the gas volume. However, it was above the ∼0.01% level of the measured threshold for methanogenesis (Cord-Ruwisch et al., 1988). There was indeed evidence of methane formation in the experiment without compost and in the Compost Maker experiments (Figure 1). We were also able to measure cellulase enzyme activity in these experiments, which indicates that cellulose degradation was ongoing, producing carbon dioxide and hydrogen (Table 3). We posit that within these 464-day experiments, syntrophic hydrogen producing fermentation reactions supplied the hydrogen and the carbon dioxide to hydrogenotrophic methanogens. The presence of the methyl co-enzyme M (mcrA) in the solid and leachate confirm the presence of methanogens in these tests. These methanogens were either endogenous to the cellulosic material itself (no compost added), or were supported by the addition of nutrients and enzymes present within the Compost Maker formulation (Figure 1). The pH in these experiments was neutral, which represent optimal conditions for methanogenesis (Wang et al., 1993).

In contrast, there was little methane formed in the Compost Accelerator experiment. Correspondingly, carbon dioxide and hydrogen levels were higher in proportion than in the other two experiments that lasted 464 days. In the Compost Accelerator experiment, the low pH likely suppressed hydrogenotrophic and acetoclastic methanogenesis (Reactions 3 and 5; Table 1). Several studies indicated that low pH inhibits both hydrogenotrophic and acetoclastic methane production (Williams and Crawford, 1984; Dunfield et al., 1993; Kotsyurbenko et al., 2004; Ye et al., 2012). The low pH in the experiment is likely the result of acetogenesis (Reaction 4; Table 1). However, we were also not able to measure cellulase activity at the end of this experiment. Therefore, it is possible that acetogenesis also ended as suggested by the pressure build-up that has more or less remained constant after day 300.

In the experiments that lasted 1965 days, carbon dioxide and hydrogen were the predominant gases. Hydrogen gas made up to 11% of the gas volume in the first 730 days of these experiments only (Figure 1). Afterwards, hydrogen decreased significantly. The presence of hydrogen within the headspace of each of the test containers after 150 and 730 days, but not at the end of the 1965 days (Figure 1), suggests that hydrogen was produced in sufficient quantity to favor acetogenesis (Reaction 4; Table 1) (Ragsdale and Pierce, 2008). Like methanogenic Archaea, acetogenic Archaea and bacteria can consume carbon dioxide and hydrogen, in this case, to form acetate (Ragsdale and Pierce, 2008; Lever, 2012; Schuchmann and Muller, 2014). Acetate formation likely explains the low carbon dioxide level at day 730, especially in the cellulose experiment. The prevalence of acetogens over methanogens is the result of acidic pH. The presence of the acyl co-enzyme A synthase (acas) gene in the solid and leachate confirms the presence of acetogens in these tests (Figure 3).

The gas pressures were relatively similar between the different experiments with the exception of the acidic experiment (Figure 1). The acidic conditions appeared to have limited the production of gases and kept gas pressure to lower levels. In addition, no cellulase activity was detected (Table 3), which indicates that cellulase was either not produced or inhibited by the acidity, thereby explaining the low gas production and resulting pressure. Juhasz et al. (2004) also reported no cellulase activity in acetic acid at pH 5. Neutral pH appears more conducive to cellulose degradation with pH of 6 yielding highest production of cellulose and highest activity (Juhasz et al., 2004).

### Implications for DGRs Isolating and Containing LILW

Evidence from our laboratory experiments suggests that if pH is maintained at neutrality in waste containers, there will be active microbial degradation of cellulose, which will produce carbon dioxide and hydrogen gas, which can then transform into methane. These conditions are predicted by typical gas models (Humphreys et al., 1997; Small et al., 2008, 2017; Avis et al., 2014). The gas generation experiment in Olkiluoto, Finland, confirms that methane is the main gas contributor emanating from low-level waste (Small et al., 2008, 2017). Methane production from the consumption of carbon dioxide and hydrogen gas limits pressure build-up. Limiting gas pressure in a DGR is desirable because it limits the possibility that gas pressure increases to a level that could create new fractures of the host rock or cement and bentonite barriers (Wragg et al., 2012). In addition, production of methane gas can maintain the pressure to equilibrium with preconstruction water pressure and keep DGR cavities dry, immobilizing water soluble radionuclides with longer half-life. In contrast, persistent pressure in equilibrium or slightly above preconstruction water pressure that maintains dry conditions would facilitate gaseous transport of C-14 and tritium gas if channels exist in the excavated damage zone between the host rock and the seal material in the shafts. However, migration of C-14 and tritium is preferred over other radionuclides soluble in water like Cl-36 and I-129 because of their short half-lives. Therefore, microbial degradation of cellulosic material and production of methane are considered ideal for the long-term isolation and containment of LILW. These processes are sustainable under circumneutral to alkaline pH (Rout et al., 2014, 2015; Bassil et al., 2015;Kuippers et al., 2015) expected in repositories excavated in rock formations with sufficient buffering capacity and backfilled with cement material.

However, some experiments in our study suggest microbial degradation of cellulosic material within waste containers will generate acid and limit gas pressure build-up. At start, the fermentation of cellulosic material contained in LILW would produce organic acids (Reaction 2; Table 1). If fermentation reactions are intense enough to lower the pH at a pH around 6 and below, hydrogenotrophic and acetoclastic methanogenesis (Reactions 3 and 5 in Table 1) would become suppressed favoring the establishment of acetogens, which would produce acetic acid and water, thereby generating more acidic leachate. The formation and build-up of acetic and organic acids inside waste containers could also limit pressure build up (Figure 1) because acidic conditions inhibit enzyme activity from the microbial degradation of cellulose (Table 3). This may also limit the accumulation of acidity in waste containers.

It is possible that acidic leachate accumulate in several waste containers within a repository. As the acidic leachate build-up inside the containers, it can slowly deteriorate the waste containers until it is punctured and the acidic leachate is released into the repository cavity. Low- and intermediate-level waste are often encased in cement within deep geological repositories (Olsson et al., 2008). The point release of acidic leachate from the different containers would likely lead to cement mineral dissolution and create advection channels in the immediate cementitious environment (Luquot and Gouze, 2009).

The length of the advection channels and whether they could reach the host rock or shaft seal would depend on the amount of acidic leachate accumulated inside each waste container but also on the gas pressure build-up inside the DGR cavity. Our experiment suggests that there is very low gas pressure when acidic conditions are observed inside waste containers. Therefore, it is possible that gas pressure remain low in DGR cavities. Low pressure would allow resaturation with meteoric water of the DGR cavities depending on hydraulic conductivity in shaft seal and host rock and the presence of fractures. If the channels extend outside of the cement barriers, the acidic leachate could be buffered by the accumulating water that bathes the cement material at pH > 12 and the host rock. Further channeling of the host rock and shaft seal material by acidic leachate would therefore be limited.

Based on this reasonable scenario, the extent of barrier degradation, in combination with mobility of water soluble radionuclides, would require additional consideration when designing a DGR. For instance, sufficient quantities of Portland Cement may be required not only in the shaft seal and as support in the DGR cavity chambers and tunnels, but as backfill into repository chambers to buffer the production of acidity from degradation of cellulose in the waste container. The importance of buffering material for the management of LILW is acknowledged by the recent results from the long-term gas generation experiment in Finland. Small et al. (2017) reported how pH has been steadily decreasing in the Olkiluoto experiment. They indicate that more Portland concrete should have been added to buffer the acid generation from the waste.

### Uncertainties: Effect of Salinity

One uncertainty of this study is if our results could be reproducible at high salinity as encountered in deep groundwater in sedimentary rock. Although this is less of an issue in granitic rock, it is possible that cellulosic degradation at high salt concentration be limited. Oren (2011) discusses that life at high salt concentrations is energetically expensive and problematic for many microbes. However, there is a growing body of evidence demonstrating that diverse populations of highly specialized or specific bacteria not only survive in hyperarid environments within halite crusts, but also manage to grow and divide (Davila et al., 2008; Wierzchos et al., 2012). More recent evidence also suggests that halotolerant microbial consortia are able to degrade lignocellulose (Cortes-Tolalpa et al., 2018). Future research is therefore recommended to first determine what would be the expected salinity of water in a DGR considering the mixing of infiltrating meteoric water and ancient groundwater and then measure microbial degradation rate of cellulosic material under such salinity.

### Uncertainties: Effect of Radiation

Our experiments were conducted in surrogate cellulosic material that did not contain any radioactivity. Radiations will be present in geological repositories but total absorbed doses and dose rates will cover a large range because of the different radionuclide inventories of waste packages, their spatial distribution and their evolution with time. Although ionizing radiation is detrimental to the survival of living organisms, a number of different mechanisms are used by bacteria to avoid lethal damage (e.g., Meike and Stroes-Gascoyne, 2000). Perhaps the best known adaptation is that of *Deinococcus radiodurans*, which achieves a high degree of radiation resistance by having multiple copies of its genome and efficient DNA repair mechanisms (Rothschild and Mancinelli, 2001). Less well appreciated is that bacteria in mineralizing environments rely on the formation of biomediated mineral coatings (e.g., silica, iron oxides) for protection against radiation (Warren and Ferris, 1998). Brown et al. (2017) recently indicated that radiation in a pond containing spent fuel may in fact stimulate iron reducing bacteria. Radiation could also enhance microbial activities by increasing the availability of electron donors. Brown et al. (2015) further reported that alteration to the metabolism of *Shewanella oneidensis* incurred in a dose response manner as a result of X-irradiation in a fuel storage pond. Irradiated *S. oneidensis* also displayed enhanced levels of poorly crystalline Fe(III) oxide reduction. Recently, MeGraw et al. (2018) reported that production of astaxanthin-rich encysted cells may be related to the preservation of the *Haematococcus* algal phenotype, potentially allowing it to survive oxidative stress arising from radiation doses associated with the spent nuclear fuel stored in pools. The oligotrophic and radiologically extreme conditions in this environment do not prevent extensive colonization by microbial communities, which play a defining role in controlling the biogeochemical fate of major radioactive species present. Therefore, it is likely that microbial activity will prevail in DGRs containing low- and intermediate-level waste which have lower dose rates than microbes experience when exposed to spent fuel in ponds. We therefore conclude that radiation from low- and intermediate-level waste would likely not change the outcome of our experiments.

## Conclusion

Long-term pressure monitoring of sealed containers and periodic analysis of the headspace gas composition showed that methane may not be the terminal gas if the acidity generated from the microbial degradation of cellulose is not sufficiently buffered to a pH above 6. Further replication with other waste types is warranted to substantiate our observations. In the meantime, predicting gas generation within a DGR safety case first requires quantification of cellulose in low-level waste. Then, a prediction of acid generation from cellulose degradation is needed along with an estimate of the localized buffering capacity of the cement and host rock. The acid generation prediction is complicated by the biodegradation of cellulosic material having multiple steps, which is rate limited by the initial breakdown of solid cellulose. The cellulose breakdown rate is also reduced by its own production of acidity because the cellulase enzyme activity is inhibited. If the cellulose in low-level waste is present in sufficient quantity to produce enough acidity to puncture the containers, the released acidic plume may dissolve the cement used as backfill and create channels. The extent of channels need to be estimated to determine if modification to DGR designs are required to prevent the migration of water soluble radionuclides.

The extent of deterioration of natural and engineered barriers caused by the generation of acidity would remain localized by excavating geological repositories several hundred meters below ground level. Siting repositories at great depth provides isolation, and additional margin of safety to counter balance uncertainties associated with the impact of acidity on natural and engineered barriers. In addition, international guidance requires demonstration that even if the shaft seal material of DGRs is severely degraded by any agent including acidity, dose to the public and the environment would remain acceptable for hundred thousands of years until the radioactive inventory decay to background levels.

Nonetheless, future research should investigate if there is enough cellulosic material in low-level waste to generate sufficient acidity to affect natural and engineered barriers. This is important to reduce uncertainty in DGR safety cases and optimize the depth of DGR hosting LILW so that people and the environment are adequately protected.

## Author Contributions

DB designed and conducted the experiments. PP was responsible for DNA analysis and primers and reviewed sections pertaining to primers and ddPCR. RG participated in the design of the experiments and provided oversight of the project and wrote the manuscript with DB.

## Conflict of Interest Statement

The authors declare that the research was conducted in the absence of any commercial or financial relationships that could be construed as a potential conflict of interest.
