# Supplementary material for: Microbial Degradation of Cellulosic Material and Gas Generation: Implications for the Management of Low- and Intermediate-Level Radioactive Waste
Source: Front Microbiol. 2019 Feb 13;10:204. doi: 10.3389/fmicb.2019.00204 (PMC6381020; doi:10.3389/fmicb.2019.00204)
Supplement: Supplementary file 1 [file Table_1.DOCX]

Table S1. Details of positive control, annealing temperature for ddPCR and the dilution of DNA.

| Target | Primer set | Positive control | T_A_ (^o^C) | 2-step or 3-step | DNA dilution |
| --- | --- | --- | --- | --- | --- |
| Bacteria | glnA F/R | glnA plasmid dil#5 or 6 | 59 | 2 | 1:10 |
| Archaea | A16s 340/806 | A16s plasmid dil#4 | 58 | 2 | 1:3 |
| Fungi | 18s euk345F/euk 499R | Arbuscular mycorrhizal Glomus irregularis, 0.1ng/µl or 0.01ng/ µl | 59 | 2 | 1:10 |
| Methanogens | mlas/mcrA | ME plasmid dil#6 | 60 | 2 | 1:3 |
| Sulfate reducers | dsr1-F-RT/dsr-R-RT | SRB plasmid dil#6 | 60 | 2 | 1:3 |
| Acetogens  dAcetyl co-A synthetase | acas For/acas Rev | Heliobacterium gDNA 22ng/ µl | 53 | 3 | 1:3 |
| Cellulase | gh61.3/61.2 | none | 54 | 3 | 1:3 |
|  | gh61.5/61.4 | none | 54 | 3 | 1:3 |
|  | bglu | none | 54 | 3 | 1:3 |
