# Supplementary figures and images for: Microbial Degradation of Cellulosic Material and Gas Generation: Implications for the Management of Low- and Intermediate-Level Radioactive Waste
Source: Front Microbiol. 2019 Feb 13;10:204. doi: 10.3389/fmicb.2019.00204 (PMC6381020; doi:10.3389/fmicb.2019.00204)

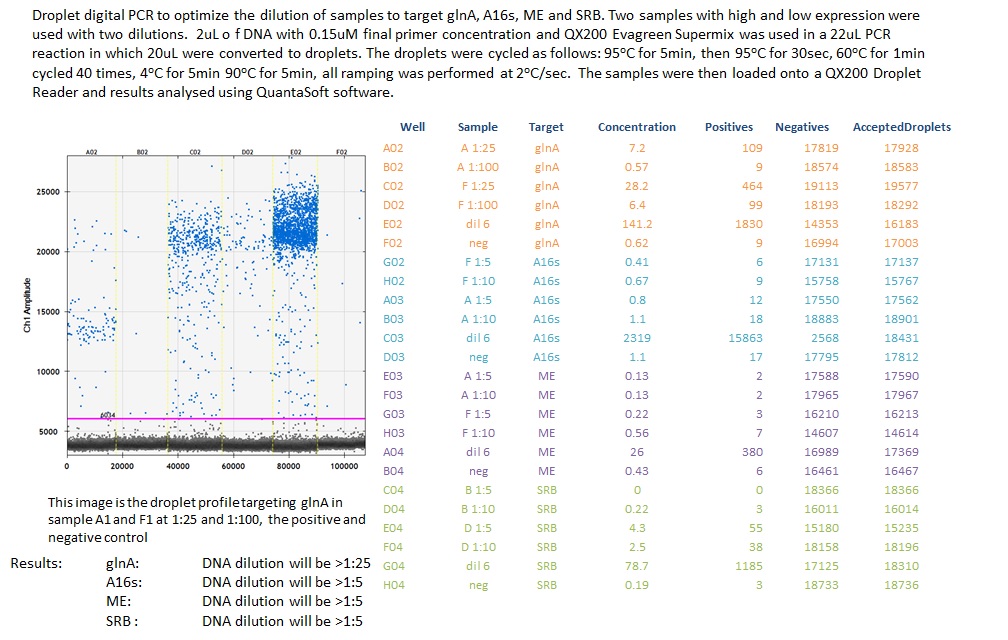

Supplement: Supplementary file 2 [file Image_1.JPEG]
